# Tissue Tracking Imaging for Identifying the Origin of Idiopathic Ventricular Arrhythmias: A New Role of Cardiac Ultrasound in Electrophysiology

**Published:** 2005-07-01

**Authors:** Sunil Roy T N, Vikram Sankar, Johnson Francis, Hiroshi Tada

**Affiliations:** * Medical College Calicut, Kerala, India; † Division of Cardiology, Gunma Prefectural Cardiovascular Center, Maebashi, Gunma, Japan

**Keywords:** Tissue tracking imaging, Ventricular tachycardia, Earliest color-coded signal, Myocardial motion amplitude, Speckle tracking

## Abstract

Several strategies for mapping ventricular outflow tract tachycardia have been reported as useful indices for differentiating between those originating from the right and the left side. Recently, tissue tracking imaging (TTI) has been demonstrated as a novel non-invasive modality for identifying the origin of outflow tract tachycardias. Tissue tracking imaging is an ultrasonographic technique that measures the myocardial motion amplitude towards the transducer in each region during systole, identifying regional myocardial displacement on the basis of myocardial velocities using color Doppler myocardial imaging principles. In this technique, the origin of the arrhythmia could be recognized as the site where the earliest color-coded signal (ECCS) appeared on the myocardium at the onset of the systole. In preliminary studies this modality was found to be useful in differentiating outflow tract ventricular tachycardias. ECCS was always found below or at the level of the pulmonary valve in all arrhythmias which could be ablated from the right ventricular outflow tract, while in those where the ECCS was above and close to the pulmonary valve it could be ablated from the left sinus of valsalva. These results indicate that TTI can provide detailed and accurate information on the arrhythmia origin of outflow tract tachycardia and may be useful for differentiating between an outflow tract tachycardia originating from the LV epicardium remote from the LSV and that from the LSV. Newer advances in echocardiographic technologies like high resolution, high frame rate real time three dimensional echocardiography with speckle tracking may further improve the precise localization of arrhythmias in the future.

Radiofrequency (RF) catheter ablation has been established as an effective and curative therapy for ventricular tachycardias (VTs) or symptomatic premature ventricular contractions (PVCs) originating from the outflow tract (OT-VT/PVCs) in structurally normal hearts [1]. Most of these arrhythmias have their origin in the septal aspect of the right ventricular outflow tract (RVOT), but some originate from the free wall of the RVOT, or from the endocardium of the left ventricular outflow tract (LVOT) [2]. Thus OT-VT/PVC can originate from several different portions of the outflow tract and RF ablation should be attempted at the optimal site of the outflow tract to cure the OT-VT/PVC. Several non invasive modalities have been used to predict tachycardia focus. However these methods have only modest diagnostic accuracy. Recently, idiopathic OT-VT/PVC originating from the left ventricular (LV) epicardium (Epi-VT/PVCs) have been reported [3]. Correct identification of the latter type of OT-VT/PVC before the RF energy applications is important in order to avoid futile RF applications from the left sinus of Valsalva (LSV) and the ensuing complications.

Several strategies for mapping RVOT VT have been used including activation mapping, pace mapping, use of basket catheter [4], and non-contact mapping systems [5]. Precordial R wave transition, QRS morphology in lead I, R wave duration and R/S amplitude of lead V1 or V2 have been reported as useful indices for differentiating OT-VT/PVCs originating from the right side from those originating from the left side [6]. However some overlap for each index was noted between these two groups especially in children [7]. Activation mapping can be used in sustained arrhythmias and pace mapping in case where clinical arrhythmia is unstable. Pace mapping and activation mapping are still the preferred methods to guide ablation in majority of cases of RVOT-VT as their effectiveness have been well proven. Non contact mapping provides a global endocardial activation map to guide ablation [8]. However RVOT is a complex and narrow three dimensional structure. There is safety concern of positioning the non contact mapping catheter in such a narrow area [9].

Algorithm based on surface ECG has overall sensitivity of 88 % and specificity of 95% for localization of OT-VT/PVCs.[6] Body surface mapping has diagnostic accuracy varying from 77% to 92%.[2] In a comparative study of non contact mapping with conventional mapping by Ribbing M et al, higher success rate was obtained by non contact mapping (100% versus 71.5%) against conventional mapping.[5] But non contact mapping was associated with higher cost, longer procedure time and Fluoroscopy time. Preliminary studies by 3 D mapping techniques have shown it to be as effective as other conventional methods for localization of OTVT/PVCs.[10]

Recently, tissue tracking imaging (TTI) has been demonstrated as a novel non-invasive modality for identifying the origin of OT-VT/PVCs [11]. TTI is an ultrasonographic technique that measures the myocardial motion amplitude toward the transducer in each region during systole, identifying regional myocardial displacement on the basis of myocardial velocities using color Doppler myocardial imaging principles [12]. It allows rapid semiquantitative visual assessment of the systolic distance of the tissue motion along the Doppler axis using a graded color display. In this technique, the origin of the OT-VT/PVC could be recognized as the site where the earliest color-coded signal (ECCS) appeared on the myocardium at the onset of the OT-VT/PVC. ECCS was always found below or at the level of the pulmonary valve, in all arrhythmias which could be ablated from the RVOT whereas the OT-VT/PVCs in which the earliest ventricular activation was recorded from the LSV had the ECCS in the myocardium above the pulmonary valve. Most of the OT-VT/PVCs in which the ECCS appeared close to the pulmonary valve could be ablated from the LSV. These results indicate that TTI can provide detailed and accurate information on the arrhythmia origin of OT-VT/PVCs and may be useful for differentiating between an OT-VT/PVC originating from the LV epicardium remote from the LSV and that from the LSV [13]. Hence TTI may be useful for identifying tachycardia origin noninvasively before the ablation procedure.

Tissue tracking imaging visualizes the velocity-time integrals (VTI) of different regions in a color, 2D mode online, together with the systolic wall motion. Tissue tracking displays the integral of the tissue velocity during systole, which equals the distance motion along the Doppler axis during systole. There are seven colour bands that indicate different distances of motion with a stepwise increase in the distance of the motion [14]. The site of the arrhythmia origin was defined as the site at which the earliest colour coded signal (ECCS) appeared in the myocardium during the first beat of the ventricular tachycardia from all of the obtained images.

In the article by Tada H et al [11], TTI was performed in 33 patients with idiopathic ventricular arrhythmias before radiofrequency catheter ablation. The ECCS during the arrhythmia was easily identified from the echocardiography by tissue tracking in all patients. The site of the arrhythmia origin, defined as the site where the ECCS appeared on the myocardium at the onset of the arrhythmia, corresponded to the site of origin as determined on fluoroscopy during activation mapping in all patients. TTI provided detailed and accurate information on the arrhythmia origin, especially in the outflow tract and facilitates catheter ablation of idiopathic ventricular arrhythmias. Radiofrequency energy delivery at the site where the earliest ventricular activation was recorded abolished 29 arrhythmias. Among the 7 arrhythmias in which ECCS were found in the myocardium above the septum, 5 arrhythmias were successfully ablated from the left sinus of Valsalva. The distance between the attachment of the pulmonary valve to the septum and the center of the ECCS (8±4 mm) in these OT-VT/PVCs with a successful ablation from the LSV was significantly shorter than that in those with a failed ablation (18±6 mm, p < 0.05) [13]. These results indicate that TTI can provide detailed and accurate information on the arrhythmia origin of OT-VT/PVCs and may be useful for differentiating between an OT-VT/PVC originating from the LV epicardium remote from the LSV and that from the LSV. However, total number of patients studied were small and they did not compare TTI with other diagnostic methods. Hence no clear conclusion could be drawn about the superiority of TTI method against other non invasive methods.

However some questions remains to be answered before it can be considered as the modality of choice. Tissue tracking imaging has limitations inherent to tissue Doppler imaging technique [15]. In the cardiac motion there are translational, rotational and deformational movements. Besides, many tissues near the heart move due to transmitted cardiac motion, vessel pulsation and respiratory motion. In addition if the initial activation occurs in a plane perpendicular to interrogating plane, tissue tracking may show tissue displacement starting from adjacent areas due to tethering movements. This issue is further complicated in patients with regional wall motion abnormalities [16]. We need to further perform the procedure during the ablation procedure to see whether the ablation site correspond exactly to the site at which ECCS were recorded. It is also unclear how successful the ablation would be in the absence of other invasive mapping techniques.

Myocardial deformation can be assessed from the B mode images by automated point wise tracking of speckle patterns. It has the advantage of angle independence and may be superior to tissue Doppler based techniques for strain estimation [17]. Whether speckle tracking imaging will be useful for identifying breakthrough points of excitation in different arrhythmias and accessory pathway localization needs to be evaluated. High resolution, high frame rate real time three dimensional echocardiography with speckle tracking imaging may be the modality for precise localization of arrhythmias in the future.

Electrical phenomena and its surrogate, the propagation of myocardial contraction, are three-dimensional events with variable sites of origin and distribution patterns. Next generation maps must be able to map the full thickness of the atrial and ventricular wall. One major challenge in clinical electrophysiology is to precisely visualize target cardiac structures and simultaneously depict electrical events. Using intracardiac ultrasound with high TDI frame rates, TDI is suitable for displaying more rapid changes in tissue motion related to the onset and propagation of electrical excitation in the myocardium at a very early stage with high temporal resolution. This ultrasound imaging technique should have an important impact on the diagnosis and management of cardiac arrhythmias. Such technology will foster simultaneous single modality visualization of anatomy, function and electrical events for the purpose of refining the proper intervention.
